# Red Cross Societies in War

**Published:** 1899-05-13

**Authors:** 


### The Hospital

A Journal of

The Medical Sciences and Hospital Administration.

Vol. XXVI.—No. 659.] Edited by Sir Henry Burdett, K.C.B., and Solomon C. Smith, M.D., M.R.C.P. [May 13, 1899.

### Red Cross Societies in War.

The announcement recently made by the War Office, that a Central British Red Cross Committee has been formed, and has been recognised by the Secretary of State for War as the official channel through which offers of voluntary aid in time of war will for the future be accepted, is a natural and very desirable outcome of the Sixth International Conference of Red Cross Societies, held at Vienna in the autumn of 1897, and reported upon by the English representative, Major W. G. Macpherson, of the Royal Army Medical Corps, whose report has been published by the Stationery Office. The experience of all our recent campaigns is amply sufficient to show that such voluntary aid would be forthcoming in time of need, not only in sufficiency to meet all reasonable requirements, but possibly even in excess of them; and that the chief necessity would be to organise and control, much more than to stimulate, the efforts of patriotism and of benevolence. Hitherto, in England, the organisation and equipment of voluntary societies have not possessed the same special significance as on the Continent, where such societies, in each State, are under the direction of a national committee of the Red Cross, known as the "Central Committee," and are elaborately organised and equipped in time of peace in order to form, under definite regulations, an integral part of the regular medical service of the army in time of war. In our country there is no such organisation in existence, and voluntary aid would come upon the military authorities in the form of a mass of chaotic and untrained elements, probably so unsuited for the actual requirements of the moment that, for a time at any rate, the working and administration of the Army Medical Service might even be considerably hampered and embarrassed. We have, it is true, certain scattered and almost competing agencies for the purpose of assisting the sick and wounded in war; but these fall far short of representing what is meant on the Continent by the organisation of voluntary aid societies under a National Central Committee of the Red Cross, the object of such being to maintain in time of peace, under the supervision and inspection of the military authorities, medical and surgical equipment, transport, and other necessaries, of the kinds and patterns approved and required by the regular Army Medical Service; to collect money for the provision of these things by voluntary subscription; to organise and equip hospitals and convalescent houses in the home territory for the reception of sick and wounded coming from the front; and to undertake many analogous duties; thus practically setting free the entire medical service for work with the army in the field. Such arrangements, as Major Macpherson points out, are easily set on foot in States where there is compulsory military service, and in which each family has a direct interest in the welfare of the sick and wounded amongst the troops, but their bearing upon this welfare is so obvious, and the importance of the right direction of effort is so plain, that we cannot doubt that the action of the War Office will be welcomed in this country by all who desire to secure the highest efficiency in military matters, and to make the best possible provision for contingencies which at some time or other are practically certain to arise.

It would be difficult to exaggerate the good effect upon public feeling which has quite lately been produced by the perfect confidence of the nation in the strength and preparedness of the navy; and a similar influence would be exerted by every measure which had a manifest tendency to increase the preparedness of the army. The committee now recognised by the War Office combines in an admirable manner all the most important elements of experience and of knowledge. The nurses of the Army Reserve are represented by Her Royal Highness Princess Christian of Schleswig-Holstein and Miss Wedgwood; the National Society for Aid to the Sick and Wounded in War is represented by Lord Wantage, V.C., Lord Rothschild, and Sir William MacCormac; St. John Ambulance Association by Viscount Knutsford and Sir John Furley,; and the War Office by the Deputy Director-General and the Assistant Director of the Army Medical Service, and by the officer in charge of mobilisation services; while Major Macpherson, to whose report, as already mentioned, the final decision of the matter must in no small degree be attributed, has been appointed secretary, and will bring to his work a very complete acquaintance with the methods which have been found most successful elsewhere. We may confidently expect that the

English organisation, framed under such auspices, will speedily attain a state of readiness to accomplish anything which can be required from it; and we hope that the official recognition of the "Red Cross" as a definite element of the medical department of the army will lead before long to a total prohibition of its employment by advertisers and other unauthorised persons.

